# Oxidative Stress and Heart Failure in Altered Thyroid States

**DOI:** 10.1100/2012/741861

**Published:** 2012-05-02

**Authors:** Pallavi Mishra, Luna Samanta

**Affiliations:** ^1^Department of Zoology, Utkal University, Odisha, Bhubaneswar 751004, India; ^2^Department of Zoology, Ravenshaw University, Odisha, Cuttack 753003, India

## Abstract

Increased or reduced action of thyroid hormone on certain molecular pathways in the heart and vasculature causes relevant cardiovascular derangements. It is well established that hyperthyroidism induces a hyperdynamic cardiovascular state, which is associated with a faster heart rate, enhanced left ventricular systolic and diastolic function whereas hypothyroidism is characterized by the opposite changes. Hyperthyroidism and hypothyroidism represent opposite clinical conditions, albeit not mirror images. Recent experimental and clinical studies have suggested the involvement of ROS tissue damage under altered thyroid status. Altered-thyroid state-linked changes in heart modify their susceptibility to oxidants and the extent of the oxidative damage they suffer following oxidative challenge. Chronic increase in the cellular levels of ROS can lead to a catastrophic cycle of DNA damage, mitochondrial dysfunction, further ROS generation and cellular injury. Thus, these cellular events might play an important role in the development and progression of myocardial remodeling and heart failure in altered thyroid states (hypo- and hyper-thyroidism). The present review aims at elucidating the various signaling pathways mediated via ROS and their modulation under altered thyroid state and the possibility of antioxidant therapy.

## 1. Introduction

 Heart failure is one of the leading causes of mortality all over the world. Heart failure (HF) is a final common pathway of various cardiovascular diseases, including sustained pressure overload (i.e., hypertension), myocardial ischemia or infarction, volume overload (i.e., valvular heart disease), and inherited or acquired cardiomyopathies. The mechanisms responsible for the progression of heart failure are emerging, and thyroid hormone-induced oxidative damage is one of them. Heart is one of the main target organs for the action of thyroid hormone, and any change in the thyroid hormone status indirectly affects the cardiac function [1]. Growing evidences have suggested that while hypermetabolic state in hyperthyroidism is associated with increase in free radical production [2, 3], the hypometabolic state induced by hypothyroidism leads to a decrease in free radical production [4]. 

Mammalian heart being an obligate aerobic organ cannot produce enough energy under aerobic conditions to maintain cellular metabolism. Thus, a constant supply of oxygen is needed to sustain cardiac functions. Oxygen acts as the terminal electron acceptor in electron transport chain and in the absence of sufficient oxygen, electron transport ceases, and cardiac energy demand is not met. However, oxygen apart from its life sustaining role also takes part in the mechanisms leading to oxidative stress. Oxidative stress is defined as the imbalance between the generation of reactive oxygen species (ROS) as a consequence of incomplete reduction of oxygen and antioxidant defense system. ROS can be induced in the heart by a variety of mechanisms such as xanthine oxidase, NAD(P)H oxidase, cytochrome P450 (CYP450), autoxidation of catecholamines and uncoupling of NO synthase (NOS), or by mitochondrial leakage. ROS formation in the heart can be induced by the action of cytokines and growth factors as well. Angiotensin II, PDGF, and TNF-*α*, for example, can induce hydrogen peroxide (H_2_O_2_) and superoxide radical (O_2_^∙−^) formation via activation of the NAD(P)H oxidase [5]. Thus, the major processes from which heart derives sufficient energy can also result in the production of ROS.

## 2. Reactive Oxygen Species and Their Metabolism

 ROS are highly reactive molecules that include free radicals, such as O_2_^∙−^ and hydroxyl (^∙^OH), which have one or more unpaired electrons, as well as compounds such as H_2_O_2_ that are not free radicals. O_2_^∙−^ is produced by single-electron reduction of molecular oxygen, and it undergoes dismutation by superoxide dismutase (SOD) (EC 1.15.1.1) to generate H_2_O_2_ which is usually broken down by catalase (EC 1.11.1.6) or glutathione peroxidase (EC 1.11.1.9) to H_2_O. The single-electron reduction of H_2_O_2_ results in the formation of highly reactive and potent hydroxyl radical (^∙^OH), via the Fenton or the metal catalysed Haber-weiss reactions. Superoxide may react with local NO^∙^ to generate peroxy nitrite (ONOO^∙^) which is itself a potent ROS. The actions of most ROS confined to the intracellular compartment of production because of their limited diffusion capacity. In contrast, H_2_O_2_ can diffuse across cell membranes and therefore has the potential to act at more distant sites [5].

Small intracellular antioxidants such as vitamin E and C, *β*-carotene, ubiquinone, lipoic acid, urate, and glutathione are categorized under nonenzymatic pathways. Glutathione also acts as reducing substrates for enzymatic activities of glutathione peroxidase. Thioredoxin and thioredoxin reductase catalyses the regeneration of a number of antioxidant molecules [6] including ubiquinone, lipoic acid, and ascorbic acid, and their absence results in developmental heart abnormalities and in cardiac death secondary to a severe dialated cardiomyopathy [7].

## 3. Biological Significance of ROS

 ROS serve an important role in several important biological processes. At physiological concentrations ROS can function as secondary messengers downstream of specific ligands including TGF-*β*1, PDGF, ATII, FGF2, endothelin, and others [8–11]. ROS also modulates the activity of specific transcription factors like NF-*κ*B and activator protein-1 (AP1) [12–15]. They also have a role in modulating inflammation and play an essential role in normal cell proliferation and growth. In addition they stimulate DNA synthesis and induce the expression of growth-related genes including c-fos, c-jun, and c-myc [16].

 Under pathophysiological conditions, dramatically elevated levels of ROS may cause significant damage to cellular proteins and membranes as well as to nucleic acids. They cause damage to cell membrane and membranes of cellular organelles by causing peroxidation of membrane lipids [17, 18]. Modification of proteins by ROS leads to inactivation and denaturation of critical enzymes [19, 20]. Introduction of strand breaks in DNA by ROS leads to mutagenesis and, thus, significantly affects gene expression [21, 22]. General ageing and age-related alterations in the cardiovascular systems have been attributed to the long-term cumulative effect of ROS [23, 24]. A number of evidences suggest the role of thyroid hormone in the generation of ROS due to its effect on the general metabolism of body.

## 4. Thyroid Hormone-Induced Oxidative Stress

 Thyroid hormone (3,3′,5-triiodothyronine, T_3_) exerts significant actions on energy metabolism, with mitochondria being the major target for its calorigenic effects. Acceleration of O_2_ consumption by T_3_ leads to an enhanced generation of ROS and reactive nitrogen species (RNS) in target tissues, with a higher consumption of cellular antioxidants and inactivation of antioxidant enzymes, thus inducing oxidative stress. A primary intracellular target for oxidative stress-induced tissue damage due to the TH is the mitochondria, the powerhouses for cellular life. Within heart mitochondria, the major locus for O_2_^∙−^ production is complex III in both euthyroid and hypothyroid states [25, 26]. Increase number of evidences point out that overall antioxidant capacity of mitochondria decreases in hyperthyroid heart, and this increases the susceptibility of mitochondria to oxidants (Figure 3).

## 5. Mechanism of Thyroid Hormone Action on Heart

**Figure 1:**
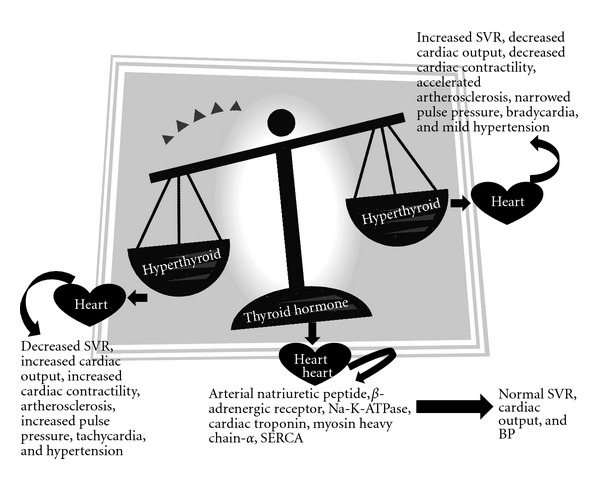
Effects of thyroid hormone on cardiac function.

TH induces changes in cardiac parameters by acting on some molecular pathways in the heart. The mode of action may be direct effect on transcription on specific and nonspecific cardiac genes (genomic effect) [27], and the other mode of action is on the action on plasma membrane, mitochondria, and sarcoplasmic reticulum (nongenomic) [28]. T_3_ and T_4_ because of their lipophilic nature can easily diffuse across the cytoplasmic membrane of cardiomyocytes. There is no evidence of conversion of T_4_ to T_3_ in cardiomyocytes [29]. Lipophilic T_3_ enters the nucleus where it binds to inactive thyroid hormone receptors (THRs) thus converting it to activated form that either homodimerizes or heterodimerizes with 9-cis-retinoic acid receptor, and the complex thus formed recognizes one of the several DNA consensus sequences and the thyroid response elements (TRE), located in the enhancer region of target genes [30, 31]. The binding of protein complex to the response elements activates the promoter of the target gene resulting in the initiation of transcription. There are a number of cardiac genes recognized as targets for transcriptional activation by thyroid hormone which include myosin heavy chain *α* (MHC-*α*), SERCA, Na-K-ATPase, *β*-adrenergic receptor, cardiac troponin I, and atrial natriuretic peptide [32–37]. On the other hand transcription of some other gene like myosin heavy chain *β* (MHC-*β*) is repressed [38]. In this regard, several lines of evidence suggest that the altered thyroid status in patients with cardiovascular disorders could modify cardiac gene expression and contribute to impaired cardiac function (Figure 1).

## 6. Heart Failure and the Role of Thyroid Hormone

 With age heart loses some blood-pumping ability, but heart failure (HF) results from the added stress of health conditions that either damage the heart or make it work too hard. A single risk factor may be sufficient to cause HF, but a combination of factors dramatically increases the risk. Heart failure itself is not a disease, but, rather, it is a syndrome that develops as the result of many conditions that damage the heart. Patients who have more than one condition face an even greater risk of developing heart failure. These conditions include (i) coronary artery disease (artherosclerosis, myocardial infarction, and ventricular remodelling), (ii) heart muscle disease (dilated cardiomyopathy, hypertrophic cardiomyopathy or inflammation or myocarditis), (iii) abnormal heart valves, (iv) heart defects present at birth (congenital heart disease) and (v) high blood pressure (hypertension) (Figure 1).

### 6.1. Coronary Artery Disease

 The heart is a muscle that works 24 hours a day. To perform well, it needs a constant supply of oxygen and nutrients, which is delivered by the blood through the coronary arteries. Coronary artery disease is the disease of the artery associated with accumulation of arethromatous plaque within the wall of the arteries that supply blood to the myocardium. Several risk factors for coronary heart disease have been well documented, including hypertension, hyperlipidemia, diabetes, a positive family story, smoking, obesity, and inactivity. However, these factors explain only in part to be attributable for cardiovascular disease. Evidence suggests that ROS may play an important role in the pathogenesis of coronary artery disease [5]. Coronary artery disease can lead to a heart attack (or myocardial infarction), which causes permanent damage to the heart muscle. In some cases, when there is a larger amount of damage, this can lead to HF. 

#### 6.1.1. Atherosclerosis

 Coronary atherosclerosis is the leading cause of death worldwide, accounting for 1/3rd of total death in industrialized countries. Atherosclerosis is a complex process involving the deposition of plasma lipoproteins and the proliferation of cellular elements in the artery wall. This chronic inflammatory condition advances through a series of stages beginning with fatty streak lesions composed largely of lipid-engorged monocyte-derived macrophage foam cells and ultimately progressing to complex plaques consisting of a core of lipid and necrotic cell debris covered by a fibrous cap. These plaques provide a barrier to arterial blood flow and may precipitate in clinical events, particularly under conditions that favor plaque rupture and thrombus formation [5].

 Oxidative stress modulates many pathological events crucial in the onset and progression of vascular disease such as oxidation of LDL, reduction of NO bioavailability, and vascular inflammation. As the process of atherogenesis proceeds, large amounts of ROS are released by inflammatory cells, as well as other constituents of atherosclerotic plaque which further facilitates atherogenesis. ROS may affect more than one fundamental mechanisms that attribute to atherogenesis like oxidation of lipids, endothelium dysfunction, proliferation of vascular smooth muscle cells (SMCs), increased adhesion of monocytes to endothelial cells, and hyperlipidemia [39].

**Figure 2:**
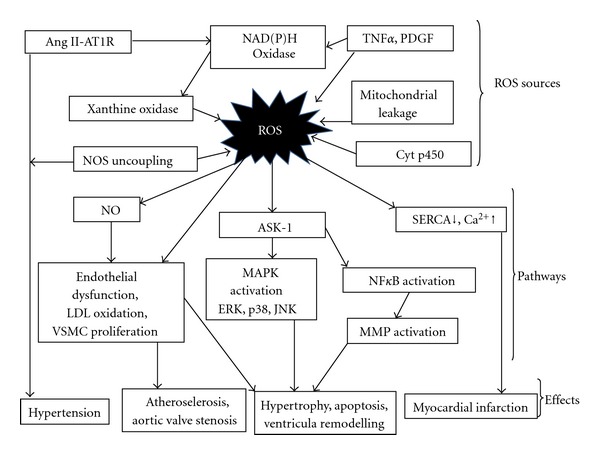
Potential sources of reactive oxygen species (ROS) and their effects on cardiac function. Cytp450: cytochrome p450, NOS: nitric oxide synthase, PDGF: platelet-derived growth factor, TNF-*α*: tumor necrosis factor, ASK-I: apoptosis-regulating signal kinase, MAPK: mitogen-activated protein kinases, NF*κ*B: nuclear factor *κ*B, MMP: matrix metalloproteinase, AngII: Angiotension II, ATIR: Angiotension I receptor, and SERCA: sarcoplasmic endoplasmin reticulum calcium ATPase.

 Oxidatively modified LDL (Ox-LDL) is a more potent proatherosclorotic mediator [40]. It causes the alteration of endothelial cells lining the arterial wall, resulting in expression of several monocytes/macrophages, which in turn release a number of growth factors [41–43]. Ox-LDL facilitates formation of matrix metalloproteins (MMPs) in vascular endothelial cells and fibroblasts. It also upregulates the expression of endothelial receptors which is responsible for the formation of foam cells, which is an early step in atherogenesis. Ox-LDL induces apoptosis in human coronary artery endothelial cells by increasing ROS production (Figure 2) [41]. 

 Endothelial dysfunction is an important early prognostic marker in the pathogenesis of atherosclerosis, contributing to plaque initiation and progression and is characterized by impaired endothelium-dependent vasodilation [44]. In addition to the above important characteristic feature of endothelial dysfunction, there is impaired synthesis, release, and activity of endothelium-derived NO which when inactivated by superoxide anion restricts its availability and results in nitrate tolerance, vasoconstriction, and hypertension as well as atherosclerosis (Figure 2) [45].

 ROS can induce vascular smooth muscle cell (SMC) growth and proliferation which is a characteristic feature of atherosclerosis. ROS increases SMC growth by stimulation of the expression of fibroblast growth factor (FGF) and fibroblast growth factor receptor-1 (FGFR-1), insulin-like growth factor-1 (IGF-1), and insulin like growth factor-1 receptor (IGF-1R) as well as epidermal growth factor receptor (EGFR) [46–49]. Evidences suggest ROS generation, via NADPH oxidase activation, plays a critical role in Ang II-induced vascular SMC proliferation, and hypertrophy in [50]. Large amount of ROS is required to induce vascular SMC death by either apoptosis or necrosis; however, this process occurs in the final stage of atherosclerosis (Figure 2) [51].

 The progression and development of atherosclerosis in humans is linked to increased adhesion of monocytes to endothelial cells. A number of studies have shown ROS upregulates the expression of various molecules in the vascular endothelium which are significant in the adhesion of monocytes to endothelial cells like that of intercellular adhesion molecule-1 (ICAM-1) [52], monocyte chemo-attractant protein-1 (MCP-1) [53], platelet endothelial cell adhesion molecule-1 (PECAM-1), and vascular cell adhesion molecule-1 (VCAM-1) (Figure 2) [52, 54].

 Hyperlipidemia is a major inducer of oxidative stress and plays a very important role in atherogenesis in susceptible animals and humans [55–57]. In hyperlipidemic patients, there is presence of abnormally high levels of LDL particles due to the enrichment of triglycerides and the high apo-B cholesterol [58]. It has been recently confirmed that ox-LDL induces proatherosclerotic NADPH oxidase expression and superoxide anion formation in human vascular endothelial cells [59], and this may be one mechanism by which ox-LDL stimulates ROS generation resulting in endothelial dysfunction as well as atherosclerosis (Figure 2). 

Hyperthyroidism and hypothyroidism represents opposite clinical conditions, albeit not mirror images. Hyperthyroidism is characterized by enhanced oxidative metabolism and markedly reduced lipid and lipoprotein plasma levels. In the hypermetabolic state characterized by hyperthyroidism, there is acceleration of free radical production, LPx in the mitochondria, and changes in the antioxidant defense system [3, 59]. Arachidonic acid content which is a polysaturated fatty acid increases in hyperthyroidism and contributed to increase in LPx. On the other hand, hypothyroidism is characterized by reduced oxidative metabolism and markedly increased lipid and lipoprotein plasma levels because of metabolic suppression caused due to lower TH [4, 60, 61]. Hypothyroid patients also show higher LPx than normo-cholesterolemic subjects which are characterized by a significant higher LDL content in the lipid peroxides and higher oxidation rate. In comparison to normal subjects, there was significant reduction in the Vitamin E content and elevation of *β*-carotene levels, which can be accounted by a blockage of *β*-carotene conversion to Vitamin E due to lack of TH [62]. As a result of this possible prooxidant activity due to elevated *β*-carotene LDL content and lack of effective antioxidant protection, the higher than normal LDL oxidation in hypothyroid patients can be explained [63]. Hypothyroid patients also suffer from hypercholestemia which is due to decreased fractional clearance of LDL by a reduced number of LDL receptors in addition to decreased receptor activity [64]. There is evidence of lowering oleic to linoleic acid ratio which is inversely proportional to oxidative stress in both the altered thyroid states and could account for the high oxidation rates (Figure 2) [65].

#### 6.1.2. Myocardial Infarction

 Myocardial infarction is one of the major conditions associated with coronary artery disease. MI occurs when blood supply to part of heart is interrupted due to occlusion of a coronary artery by thrombosis following the rupture of a vulnerable atherosclerotic plaque, which is an unstable collection of lipids and white blood cells (especially macrophages) in the wall of an artery [39]. The resulting ischemia and oxygen shortage, if left untreated for a sufficient time, can cause damage and/or death (infarction) to myocardium. Myocardial infarction results in the migration of macrophages, monocytes, and neutrophils into the infarct zone which in turn generates an inflammatory response by initiating intracellular signaling and neurohormonal activation. Inflammatory response is associated with increased generation of ROS which in turn contributes to the formation of ox-LDL leading to the development of atherosclerotic plaque and augmentation of MMPs leading to vessel plaque rupture, coronary thrombosis, and occlusion (Figure 2) [5].

MI is usually initiated by myocardial ischemia as a result of narrowing of arteries during artherosclerosis and ROS are generated, after reperfusion. Myocardial ischemia is suggested to affect contractility of myocardium as a consequence of cell death by either necrosis or apoptosis. However, if the coronary flow is not restored within a critical period of time, reperfusion itself may cause a wide variety of harmful effects in the ischemic heart a phenomenon referred to as ‘“reperfusion injury”' [66–71]. Ischemic injury is associated with alterations in myocardial metabolism, including depletion of energy stores while reperfusion injury is associated with additional changes, including the development of oxidative stress and the occurrence of intracellular Ca^2+^ overload. The initial process contributing to ischemic injury is a reduction of cellular energy status as a result of compromised mitochondrial production of ATP via impaired oxidative phosphorylation and electron transport due to reduced delivery of oxygen and substrates to the myocardium. In spite of the increase in anaerobic glycolysis in ischemic hearts, this metabolic change is not sufficient to meet the high-energy demands for maintaining cardiac function [66, 70, 72]. Mitochondrial electron transport system derangement also promotes the generation of oxyradicals and development of oxidative stress in the ischemic heart [68, 73]. Myocardial ischaemia either with or without reperfusion induces ROS and inflammatory cytokines. Inflammatory cytokines, for example, tumour necrosis factor-*α* (TNF-*α*), IL-1b, and IL-6 are produced as a host reaction in the ischaemic region and surrounding myocardium, and this activate is the MMPs and collagen deposit which contribute to the structural changes and tissue repair of injured myocardium [74]. 

Ischemia reperfusion has been considered model of oxidative injury to test the view of reduced capability of hyperthyroid tissues to face oxidative damage. It has been found that during reperfusion following 20 minutes ischemia, hyperthyroid hearts displayed significant tachycardia which results in decline in antioxidant capacity and is associated with the reduced capacity of the hyperthyroid heart to face an oxidative stress [75]. There are instances of wider range of oxidative damage to lipids and proteins parallel to a higher rate of H_2_O_2_ production due to ischemicreperfusion in mitochondria from hyperthyroid hearts than in those from euthyroid hearts (Figure 2). In addition the concentration of important liposoluble antioxidants like ubiquinols and Vitamin E significantly reduces after an episode of ischemiareperfusion in hyperthyroid hearts which could lead to increase in the extent of mitochondrial dysfunction and tissue impairment following damaging action of nitric oxide. The decline in mitochondrial respiration could also be due to combination of oxidative stress and the increase in Ca^2+^ concentration that occurs in myocardial cells during ischemiareperfusion [75]. In presence of Ca^2+^ oxidative alternations of mitochondrial inner membrane protein thiols leading to mitochondrial permeability transition (MPT) that leads to mitochondrial swelling [76].

 Calcium ions are essential for cardiac contractions and relaxation. ROS act as cardiodepressant through impairment of Ca^2+^ homeostasis through direct effects on membrane proteins involved in the regulation of cation transport and LPX, which causes a change in membrane permeability. They cause intracellular Ca^2+^ ion influx mediated by increased membrane LPX and opening of voltage-sensitive Ca^2+^ channels or Na^+^/Ca^2+^ exchanger leading to accumulation of intracellular Na and Ca ions (Figure 2) [77, 78]. Specifically, ischemia reduces the activity of sarcolemmal Na/K-ATPase, increases the activity of the Na^+^-H^+^ exchanger, and promotes the activation of the Na^+^-Ca^2+^ exchanger in a reverse mode. Calcium transport is also affected by ischemiareperfusion because oxidative stress adversely influences Ca^2+^ handling proteins in the sarcoplasmic reticulum (Ca^2+^ pump, sarcoplasmic reticulum Ca^2+^-ATPase, and the Ca^2+^ release channel) and sarcolemma (sarcolemmal Ca^2+^,pump and the L-type Ca^2+^ channels) and thus contributes to the development of an intracellular Ca^2+^ overload [71, 79]. The pathological effects induced by intracellular Ca^2+^ overload are mediated by Ca^2+^-induced activation of membrane phospholipases and proteases. ROS depress the activity of sarcoplasmic reticulum Ca^2+^ ATPase (SERCA2) a membrane calcium pump, which plays a crucial role in calcium handling and a determinant of myocardial contractility [80].

 Thyroid hormone regulates expression of specific cardiomyocyte genes independent of its effects on cardiac growth and protein synthesis. Thyroid hormone critically regulates cardiac performance since several genes encoding important structural and regulatory proteins in the myocardium, including myosin isoform expression, calcium-cycling proteins, and protein kinases (such as PKC*α* and PKC*ε* known to phosphorylate cardiac contractile proteins) are thyroid hormone responsive. Certain T_3_-responsive cardiac genes, the myosin heavy chains (MHC), phospholamban (PLB), and sarcoplasmic reticulum calcium-activated ATPase (SERCA2) are important determinants of cardiac contractility [81]. The performance of SERCA is influenced by the level of expression of phospholamban which is inversely proportional to SERCA activity. Low expression of phospholamban and higher expression of SERCA are responsible for calcium uptake into lumen of sarcoplasmic reticulum and thus regulate myocardial relaxation during diastole. It has been found that TH upregulates expression of SERCA and downregulates expression of phospholamban, thereby enhancing myocardial relaxation [82]. Thyroid hormone not only promotes the phosphorylation of phospholamban, on SERCA but also effects the expression of other ion channels such as Na^+^/K^+^-activated ATPases, Na^+^/Ca^2+^ exchanger and some voltage-gated K^+^ channels, thereby coordinating the electrochemical and mechanical responses of the myocardium [83, 84]. Low T_3_ state after acute MI may contribute to the changes in cardiac specific gene expression and further compromise the cardiac myocyte in its response to the ischemic injury. In hypothyroidism, there is significant increase in PLB content and decrease in SERCA level in the heart resulting in decreased SR Ca^2+^ uptake parameters [85, 86]. Treatment with T_3_-promoted phosphorylation of PLB, thereby decreasing its inhibitory action on sarcoplasmic reticulum calcium uptake and improving LV function. Therefore, it is plausible that, as a result of the low serum T_3_ after acute MI, cardiac PLB was similarly altered and that T_3_ replacement improved myocyte contractility through this pathway. On the other hand, hyperthyroidism results in increase in SERCA and decrease in PLB levels results in higher number of SR Ca^2+^ pumps leading to increased uptake of SR Ca^2+^ and thus hasten cardiac relaxation as compared to euthyroid hearts [87]. Thus, hypothyroid and hyperthyroid hearts are associated inversely with each other in the levels of SERCA, and PLB to the alterations in contractile parameters.

#### 6.1.3. Ventricular Remodeling

 Myocardial infarction (MI) is followed by a very important structural event that is left ventricular (LV) remodeling. MI results in acute loss of myocardium and is frequently followed over weeks and months by a series of alterations in cardiac structure, function, geometry, and volume collectively known as cardiac remodeling [88, 89]. Cardiac remodeling plays an important role in the progression of cardiovascular diseases including myocardial infarction, valvular heart diseases, myocarditis, and dilated cardiomyopathy. In addition to cardiac myocytes, fibroblasts, extracellular matrix proteins, and coronary vasculature are also involved in the remodeling process. Cardiac remodeling is associated with alterations of many mediators such as neurohumoral factors, cytokines, enzymes, ion channels, oxidative stress, and mechanical stress. Although remodeling is initially an adaptive response to maintain normal cardiac function, it gradually becomes maladaptive and leads to progressive decompensation. Growing evidence suggest that ROS are involved in the pathophysiology of myocardial remodeling and failure. The mechanism involved in left ventricular (LV) remodelling is multifactorial and involves many biological processes which are related to each other like ischaemia, oxidative stress, inflammatory cytokines, activations of matrix metalloproteinases (MMPs), mechanical stress, hypertrophy, and apoptosis/necrosis. Left ventricular (LV) remodeling ultimately elicits LV dilatation accompanied by LV dysfunction and thus could be one of the most important causes of MI [88, 89]. The following paragraph summarizes the recent data on the events and pathways during ventricular remodeling with special reference to oxidative stress.

 ROS has also a role in the process underlying cardiac remodeling by modulating extracellular matrix function via effects on fibroblast proliferation and collagen synthesis, which may in turn activate MMPs, levels of which are increased following MI. Sustained MMP activation might influence the structural properties of the myocardium by providing an abnormal extracellular environment with which the myocytes interact, leading to development of left ventricular remodeling and failure. After MI, MMPs, and tissue inhibitors of MMPs (TIMPs) have a major contribution in cardiac repair and LV remodelling. MMPs constitute a family of endopeptidases having zinc at their active site, dependency on Ca^2+^ for their activity, and an ability to react with specific tissue inhibitors (TIMPs) to form enzymatically inactive complexes [90]. A balance between MMP and TIMP activities is a prerequisite for normal function of an array of physiologic processes, and disruption of this balance may result in diseases associated with uncontrolled turnover of extracellular matrix (ECM). It is also reported that inflammatory cytokines and ROS mediate MMP induction or stimulation and decrease TIMP levels and collagen synthesis [91]. Structural changes of myocardial tissue are elaborated by sustained induction of inflammatory cytokines. The triple-helical structure of collagen renders it resistant to proteolytic degradation, except by MMPs, which are secreted into the extracellular matrix in their latent proenzyme form. Collagenolytic activity is confined to regions of injury by TIMPs. These low-molecular-weight proteins form high-affinity complexes with activated MMPs and neutralize collagen degradation by blocking the catalytic domain of MMPs. The synthesis of TIMPs is modulated by the levels of activated MMPs, such that collagen degradation reflects the disequilibrium between MMPs and TIMPs (Figure 2) [92].

It is now recognized that cardiac dysfunction after acute myocardial infarction (AMI) is due to the extent of remodeling of viable myocardium. Thyroid hormone signaling probably plays an important role in pathophysiology of cardiac remodeling at later stages after myocardial infarction. However, thyroid hormone administration shortly after left coronary ligation displayed striking effects on cardiac remodeling and consequently on cardiac function. In fact, in viable myocardium, the expression of *α*-MHC and the ratio of SERCA/PLB were increased while ß-MHC expression, was decreased [93]. Furthermore, PKC*α* expression was significantly reduced while PKC*ε* expression further declined, both of which are regulated by thyroid hormone [94]. During this process, a fetal-like phenotype is recapitulated including a shift to fetal pattern of myosin isoform expression with an increase in *β*-MHC and decrease in  *α*-MHC expression and TH is a major contributor to this process by changing the thyroid hormone-thyroid hormone nuclear receptors axis [93, 95, 96]. Cardiac performance is critically regulated by thyroid hormone because several genes encoding important structural and regulatory proteins in the myocardium, like myosin isoform expression, calcium-cycling proteins, and protein kinases (such as PKC*α* and PKC*ε* known to phosphorylate cardiac contractile proteins) are thyroid hormone responsive [94, 97–100].

Loss of contracting myocardium due to MI results in a chronic increase in the work load of the remaining viable myocardium. The heart responds with an increase in muscle mass, and this process of heart hypertrophy represents a fundamental compensatory mechanism that permits the ventricle to sustain normal perfusion pressure. In response to this pressure overload left ventricular (LV) hypertrophy is the primary remodeling process in the myocardium. There is strong evidence implicating the role of ROS signaling in the genesis of cardiac hypertrophy. Complex and interesting interactions occur between cardiac hypertrophy induced by excess thyroid hormone action and cardiac hypertrophy occurring with heart failure. The thyroid hormone-mediated cardiac hypertrophy in its initial phases presents a physiological hypertrophy with increases in SERCA2 levels and decreased expression of *β*-MHC. In contrast, pressure overload-induced heart failure leads to a “pathological” cardiac hypertrophy which is largely mediated by the activation of the calcineurin system and the MAP kinase-signaling system responsive [94, 97–100]. 

 Although the biological mechanisms for progression and ventricular remodeling have no definite explanation, mounting evidence supports the theory that ventricular dysfunction worsens due to myocyte apoptosis as a consequence of increased ROS formation [101]. It has been demonstrated that a new signaling molecule, p66shc, which links oxidative stress and apoptosis is upregulated in HF. p66shc is an oxidant stress-induced, proapoptotic protooncogene known to be activated by phosphorylation in response to stimuli such as H_2_O_2_, UV radiation, or epidermal growth factor. Transgenic disruption of p66shc or inactivation of its ability to be phosphorylated confers resistance to oxidative damage and prolongs life in mice [102]. Ask1, a 160 kDa serine/threonine protein kinase, is an ROS-sensitive MAP kinase and is responsible for activation of both p38 and Jnk pathways [103]. Ask1 is ubiquitously expressed in most mammalian cells is activated by many stress signals like ROS and proinflammatory cytokines including TNF-*α*. G-protein-coupled receptors agonist e.g. nor-epinephrine, angiotensin II, and endothelin also activate Ask1 via ROS generation. Ask1 is also involved in NF-*κ*B activation and cardiomyocyte hypertrophy via G protein-coupled receptor [13]. Overexpression of Ask1 induces apoptosis in cardiomyocytes. It has been observed that signaling pathways link myocardial apoptosis with cellular ability to cope with oxidative stress.

 Increase in apoptotic rates and DNA laddering was detectable in T_4_-treated rat hearts. Angiotensin II further increased the apoptotic rate of hyperthyroid hypertrophied cardiomyocytes. The apoptotic rate in cardiomyocytes was dependent on the extent of cardiomyocyte hypertrophy and also susceptibility to apoptotic stimulation by angiotensin II. Depressed cardiac contractility and enhanced apoptosis may lead to heart failure in hypertrophied hyperthyroid myocardium [104]. Very few evidences regarding the progression of apoptosis in hypothyroid heart is present which open the door for further investigation in this field.

### 6.2. Cardiomyopathy

Cardiomyopathy, which literally means “heart muscle disease," is the deterioration of the function of the myocardium leading to risk of arrhythmia or sudden cardiac death or both. Cardiomyopathies are now defined as diseases of the myocardium associated with cardiac dysfunction and classified by the dominant pathophysiologic factors. These include dilated cardiomyopathy (dilation of left ventricle), hypertrophic cardiomyopathy (abnormal thickening of heart muscle), restrictive cardiomyopathy (heart muscle becomes rigid and less elastic), arrhythmogenic right ventricular cardiomyopathy, and specific cardiomyopathy. The term “specific cardiomyopathy” is now used to describe heart muscle diseases that are associated with specific cardiac or system disorders. The condition may also be caused by diseases elsewhere in the body that affect the heart, such as amyloidosis, a rare condition in which abnormal proteins present in the blood are deposited into the heart.

It has been determined that there is increase in oxidative stress in patients with dilated cardiomyopathy by proving that erythrocytes and erythrocyte membranes from these patients were more prone to LPX and oxidative damage than those from healthy controls. These results show that antioxidant defense of erythrocytes in patients with dilated cardiomyopathy is weak compared with that in healthy controls. Patients with dilated cardiomyopathy have impaired myocardial contractility and can have fatal ventricular arrhythmias. Membrane changes due to increased oxidative stress may be partly responsible for these manifestations of the disease although ROS cannot be totally responsible for the particular disease. Although peroxidation of membrane lipids is a relatively slow process, however, it increases LPX due to recurring ischemia-reperfusion cycles in the heart and skeletal muscle, catecholamine autoxidation, and changes in membrane proteins. In this respect*, in vitro* studies have shown that oxidative stress induces oxidation of SH groups and can impair Ca^2+^-ATPase activity of sarcoplasmic reticulum [105], and Na^+^ K^+^-ATPase of plasma membranes [106]. Moreover, alterations of hormone receptors containing a critical SH moiety, involved in maintaining calcium homeostasis, results in intracellular calcium overload and hence contribute to the mechanism of contractile dysfunction and ventricular arrhythmias in dilated cardiomyopathy. Release of these ROS due to conversion of xanthine dehydrogenase to xanthine oxidase during purine degradation may peroxidize the cell membrane [107]. It has been demonstrated that myocardial high-energy phosphate concentrations were reduced in patients with dilated cardiomyopathy [108]. Activity of creatine kinase (EC 2.7.3.2), which plays an important role in rapid resynthesis of ATP when the heart increases its work in myocardial and skeletal muscle in case of high-energy phosphate metabolism, is also impaired as a result of ROS-induced SH group oxidation at the active site (Figure 2). It has been found that inhibition of the reaction of creatine kinase decreases the contractile reserve of the isolated rat heart-dilated cardiomyopathy [109]. Nevertheless, a relative deficit in antioxidant reserves may contribute to cardiac failure [110]. Therefore, exogenous administration of antioxidants may slow the progression of cardiac abnormalities and may decrease the incidence of life-threatening ventricular arrhythmias or sudden death in dilated cardiomyopathy or, generally, in congestive heart failure.

Reversible cardiomyopathy has been associated with endocrine dysfunction including thyroid dysfunction although extensive work in this regard has not been carried out. Excess thyroid hormones can lead to cardiac disease through the potential mechanism of thyrotoxicosis which is associated with high cardiac output failure. Hyperthyroidism results in tachycardia and atrial fibrillation which have been reported as causes of cardiomyopathy (Figure 1) [111].

### 6.3. Myocarditis

Cardiomyopathy can sometimes develop after exposure to a virus. Viral myocarditis is usually a self-limited disease but may occasionally lead to severe cardiac damage and heart failure. Cardiac damage in coxsackievirus myocarditis involves not only apoptotic but also necrotic cardiomyocyte death [112–117]. Increase in apoptosis and reduction in plasma GSH levels in cardiomyocytes of AVM patients suggests the role of ROS in the induction of cardiomyocyte apoptosis during CVB3 myocarditis. It has been reported that GPx knockout mice with reduced antioxidant capabilities develop more severe myocarditis when infected with CVB3 as compared to wild-type mice [118]. Serious pathological aggravation of oxidative stress and severe disturbance of nitric oxide metabolism has been found in patients suffering from acute viral myocarditis (AVM). The inflammatory cytokines particularly interleukin-I (IL-1) are released by phagocytic cells like lymphocytes, neutrophils, granulocytes, and macrophages in the inflammatory reaction in the cardiac muscle cells and tissues as a result of oxidative stress. This in turn activates iNOS which releases large amount of NO which combines with superoxide anion radical to produce ONOO^−^, damaging cell functions and deactivating the antioxidases such as superoxide dismutase, catalase, and glutathione peroxidase, by combining with their hydrosulfide group (-SH) [119, 120]. Moreover, excessive nitric oxide is rapidly oxidized into nitrogen dioxide, which acts as catalyst in the LPx of polyunsaturated fatty acids, thus peroxidizing the cell and tissue membranes. In addition to this, the significant decrease in the synthesis or regeneration of glutathione peroxidase resulting in loss of GPx activity of removing lipoperoxide. All these mechanisms might be responsible for the significant increase in the level of lipoperoxide in the AVM patients' bodies [119–122]. Additionally, the AVM patients appear to have poor appetite because of their temperature elevation resulting in reduced intake of dietary antioxidants (such as vitamin C, vitamin E, and *β*-carotene). This leads to a significant decrease in the antioxidants levels in their bodies which disrupts the dynamic imbalance between oxidation and antioxidation, resulting in pathological aggravation of a series of free radical chain reactions in the patients' bodies. 

Graves' disease and/or hyperthyroidism has been found to be associated with myocarditis. Evidence suggests that there is an association between lymphocytic myocarditis and lmphocytic thyroiditis. On the other hand, hypothyroidism and Hashimoto's thyroiditis have never been reported in association with myocarditis as a cause of sudden death [123–127]. The reason why myocarditis has been found exclusively in hyperthyroidism and Graves' disease and not in hypothyroidism and Hashimoto's thyroiditis is still a matter of debate. One explanation may be that the symptoms of myocarditis may be due to autoimmune myocarditis rather than a viral infection.

### 6.4. Valvular Disease

 Heart valve problems can result from disease, infection (endocarditis), or a defect present at birth. When the valves do not open or close completely during each heartbeat, the heart muscle has to pump harder to keep the blood moving. Heart failure results when the workload becomes too great. Heart valves are composed mainly of extracellular matrix, smooth muscle cells, fibroblasts, and endothelial cells [128].

 “Degenerative" aortic valve stenosis (AVS) is a valve disease. It increases with advancing age in comparison with rheumatic diseases, which prevail in younger populations. Degenerative and rheumatic HVD is caused by several risk factors such as genetic, inflammatory, autoimmune, infectious, and oxidative stress [129]. Calcified aortic valve disease ranges from mild aortic valve sclerosis to severe aortic valve stenosis. It is a regulated disease process and shares pathophysiological features with atherosclerosis. Histological analysis of valve lesions showed similarities to atherosclerotic plaque evolution, such as the presence of lipoproteins, foam cells, macrophages, T lymphocytes, extracellular matrix proteins, heterotopic calcification, and bone tissue. These tissue factors may be the result of “active" biologic processes of chronic inflammation and tissue repair, sustained by angiogenesis similar to the processes involved in atherosclerosis. Fibrosis and calcification are characteristic features of degenerative aortic valve lesions [130]. The calcified aortic valve lesion develops endothelial injury and is characterized by chronic inflammation [131], lipoprotein accumulation [132], renin-angiotensin system activation [133], and fibrosis. Coronary atherosclerosis and valvular sclerosis are similar processes, and they may occur coincidentally [134]. Calcified lesions in stenotic aortic valves resemble atherosclerotic lesions and contain calcium, oxidized low-density lipoproteins, areas of neovascularization, high levels of matrix-remodeling enzymes, and apoptotic cells within the valvular plaque. Oxidative modification of low-density lipoprotein by ROS has been reported to influence initiation and progression of valve lesions [132]. The role of oxidative stress and systemic inflammation in rheumatic HVD is well established [130]. In contrast to atherosclerotic lesions, increased oxidative stress seems to be partly due to reductions in antioxidant enzyme expression and activity [130]. In addition, NOS uncoupling might play an important role in the generation of superoxide in calcified aortic valves leading to the pathophysiology of degenerative AVS (Figure 2).

The possible linkage of thyroid dysfunction and valve sclerosis has not been investigated so far although it may be regarded as pathophysiologically plausible. However, a possible association between thyroid dysfunction might at least explain the elevated cardiovascular mortality reported in thyroid dysfunction, taking into consideration that AVS and MAC (mitral annular calcification) are markers of increased cardiovascular mortality.

### 6.5. Hypertension

In high blood pressure (BP), the pressure and resistance in the body's arteries is increased. Uncontrolled high blood pressure increases the risk of developing heart failure by two to three times. When pressure in the blood vessels is too high, the heart has to pump harder than normal to keep the blood circulating. This takes a toll on the heart, and overtime the chambers get larger and weaker. Oxidative stress may contribute to the generation of hypertension by various mechanisms which includes quenching of the vasodilator nitric oxide (NO) by ROS [135] generation of vasoconstrictor lipid peroxidation products, such as F2-isoprostanes [136] depletion of tetrahydrobiopterin (BH4), an important NO synthase (NOS) cofactor [137] as well as structural and functional alterations within the vasculature [138]. Oxidative stress promotes vascular smooth muscle cell proliferation and hypertrophy and collagen deposition, leading to thickening of the vascular media and narrowing of the vascular lumen. In addition, increased oxidative stress may damage the endothelium and impair endothelium-dependent vascular relaxation and increases vascular contractile activity. All these effects on the vasculature may explain how increased oxidative stress can cause hypertension.

 There are a number of possible sources of free radical production within the vasculature which include NADPH oxidase, NOS, cyclooxygenases, lipoxygenases, and xanthine oxidase, all of which are functional in endothelial cells. During the metabolism of arachidonic acid by CYP450, an important cofactor is the NADPH oxidase system, superoxide, hydrogen peroxide, and hydroxyl radicals are generated. Cytochrome 450 2C9 isoform is a functionally significant source of ROS in coronary arteries has shown by *in vitro studies* (Figure 2). These studies indicate that CYP450 plays an important role in endothelial dysfunction and hypertension by production of oxidative stress within the vessel wall [139]. One important endogenous regulator of CYP450 metabolism is thyroid hormone, T3, which not only contributes to the regulation of certain hepatic CYP450 enzymes but is also a key determinant of the expression of CYP450R in other tissues.

 Human studies have not been as consistent as animal studies and results vary depending on the marker of oxidative damage being investigated. Studies using nonspecific markers of oxidative damage have observed higher superoxide and hydrogen peroxide production in individuals suffering from hypertension, which returned to basal levels following reduction in BP [140]. Reductions in SOD and glutathione peroxidase activity have been observed in hypertensives with no history of early treatment when compared with controls which shows that SOD activity was inversely correlated with reduction BP within the hypertensive group, but not controls [141]. There is a significant correlation between hydrogen peroxide levels, and systolic BP as higher production of H_2_O_2_ has also been observed in treated and untreated hypertensives compared with normotensives [142]. An imbalance in superoxide and nitric oxide production may account for reduced vasodilation, which in turn can favor the development of hypertension. *In vitro* and in human studies support this hypothesis (Figure 2).

 Regarding hypertension, endothelial cells play a major role in arterial relaxation by releasing NO in the vasculature. Nitric oxide causes vascular relaxation [143]. Nitric oxide synthase (NOS), and in particular the endothelial isoform of NOS (eNOS), is now recognized as an important source of O_2_^·−^ which is a major determinant of NO biosynthesis and bioavailability [144, 145]. It has been found that eNOS can generate superoxide instead of NO in response to atherogenic stimuli. This led to the concept of “NOS uncoupling,” where the activity of the enzyme for NO production is decreased, in association with an increase in NOS-dependent superoxide production [146] leading to a dramatic increase in peroxynitrite formed by the NO-superoxide reaction. This has additional harmful effects on vascular function by oxidation of cellular proteins and lipids [147].

Recently it has been reported that vascular endothelium is a target of TH and an excessive endothelial NO production plays a role in vasodilation in hyperthyroidism. In addition, increased vascular NO production has been reported in rats in hyperthyroid states or after treatment with T_3_ [148]. Nitric oxide (NO) production by VSMCs has been considered as one of the causes of overproduction of NO in the vascular wall and might be responsible for the local control of vascular function [149]. A previous study demonstrated that hyperthyroidism reduces both vasoconstriction and blood pressure elevation induced by catecholamines, which also involves NO generation [150]. Thyroid hormone acutely stimulates relaxation of VSMCs by increasing production of NO which occurs rapidly by the activation of iNOS and nNOS via mechanism of the PI3K/Akt-signalling pathway [151–153].

Increasing number of evidences indicates that NADPH-driven generation of ROS and activation of reduction-oxidation (redox)-dependent signaling cascades are involved in the role of Angiotensin II-induced hypertension [154] documented in cardiomyocytes, vascular and endothelial cells. There is an increase in Ang II-dependent filtration of circulating neutrophils into myocardial tissues and which may be a source of ROS by increasing expression of inflammatory cytokines. Ang II elicits its actions via two distinct receptors: the Ang I receptors (AT1R) and Ang II receptors (AT2R) which are ideal candidates for maintaining a proper balance between NO and ROS [154, 155]. It has been demonstrate that Ang II, acting through the AT1 receptor, stimulates nonphagocytic NADPH oxidase, causing the accumulation of superoxide, hydrogen peroxide, and peroxynitrite produce an inflammatory response in pathophysiological conditions (Figure 2). Blockage of AT1R will stimulate the AT2R and oppose the effect of AT1R activation, a mechanism that appears to be involved in the beneficial effects of the angiotensin receptor blockers. Collectively, these results indicate that chronic loss of angiotension-converting enzyme (ACE2) leads to AT1R -mediated NADPH oxidase activation, increased oxidative stress, neutrophil recruitment, and MAPK activation through GCPR-signalling ultimately leading to adverse ventricular remodeling and heart disease [156]. 

Thyroid hormone downregulates AT1R mRNA expression at both transcriptional and posttranscriptional levels. Evidences showed that vascular endothelium is a target of thyroid hormone, and an excessive endothelial NO production plays a role in vasodilation in hyperthyroidism [157]. It has been reported that NO donor inhibited AT1R expression in VSMCs [158]. Therefore, it may be possible that T_3_ indirectly inhibits vascular AT1R expression through the production of NO in addition to direct downregulation of AT1R. Thyroid hormone increases the basal metabolic rate in almost every tissue and organ in the body, and increased metabolic demands lead to changes in cardiac output, (systemic vascular resistance) SVR and BP. Hyperthyroidism has been reported as a secondary cause of isolated systolic hypertension, which is the most common cause of hypertension, and can be reverted back to normal by treatment of hyperthyroidism and use of *β*-blockade. Hyperthyroidism also causes diastolic hypertension by causing endothelial dysfunction, impaired VSM relaxation which leads to increased SVR and can also be reverted back by thyroid hormone replacement. Thyroid hormone causes resistance in peripheral vasculature through a direct effect on VSM and decreased mean arteriole pressure, and this change is sensed by the juxtaglomerular apparatus of kidneys which is volume and pressure sensitive. This leads to the activation of the rennin-angiotension system and increase in rennin secretion. This is followed by a cascade of events including increased levels of angiotensin I and II, angiotension converting enzyme (ACE) and aldosterone while increasing blood volume and preload and thus contributes to characteristic increase in cardiac output and BP [159]. In hyperthyroidism the combined effects of decreased SVR, increased heart rate, and blood volume effects increase cardiac output 50–300% higher than in normal individuals. On the other hand in hypothyroidism it is just opposite and cardiac output may decrease by 30–50% [160]. Hypothyroidism is accompanied by a rise in diastolic BP because of low cardiac output and narrow pulse pressure. There are various evidences suggesting the role of natriuretic peptides in regulation of salt and water balance and thus in regulation of BP. Thyroid hormone affects the expression of the prohormone genes for each natriuretic peptide which is altered with changes in thyroid disease state [161].

### 6.6. Congenital Heart Disease

In the heart and its chambers if incorrectly formed, the healthy parts have to work harder to make up for it. Oxygen-free radicals are incriminated in the causation of several congenital diseases. Oxidative stress, due to ROS and RNS, may contribute to phenotypic changes in infants hearts adapted to chronic hypoxia and to the pathogenesis of myocardial injury during both ischemia/reperfusion and hypoxia/reoxygenation (Figure 2). Cardiovascular disease is a significant cause of death and chronic illness in childhood. There is scarce data in literature regarding the association between congenital heart disease and oxidative stress in children. Children with congenital heart disease have poorer clinical outcomes because hypoxia in these children reduces the antioxidant reserve capacity leading to a greater susceptibility to the oxidative stress [162]. The potential contribution of oxidative stress to cardioprotection in infants induced by adaptation to chronic hypoxia and by ischemic preconditioning is poorly understood. Endothelial nitric oxide synthase (eNOS) protein and its product nitric oxide are increased in chronically hypoxic infant hearts to protect against ischemia. Thus, eNOS appears to be critically important in adaptation of infant hearts to chronic hypoxia and in resistance to subsequent ischemia by regulating the production of reactive oxygen species [163]. Infants and children also suffer from some important thyroid hormone-related heart disease which in most cases is a side effect of thyroid disorder in the mother. Maternal illnesses play a significant role in the development of heart defects in fetuses. Although the embryo does not have the disease, prolonged exposure to metabolites of the maternal illness leads to the development of congenital malformations. In case of hyperthyroid mothers, thyroid-stimulating immunoglobulin can cross the placental barrier and stimulate the fetal thyroid gland, causing neonatal hyperthyroidism leading to conditions like tachycardia, bounding pulses, systolic hypertension, a systolic murmur, and sometimes congestive heart failure, in infants. If treatment for neonatal hyperthyroidism is delayed or inadequate, potentially fatal arrhythmias and cardiac failure may develop. Exposure to iodine might occur during cardiac catheterisation or surgery and may induce a transient form of hypothyroidism in infants [164]. Evidences indicate that the heart drug amiodarone, which is an extremely effective treatment for infants and children with tachyarrhythmias may also cause hypothyroidism as a side effect [165].

### 6.7. Epilogue

 Oxidative stress is one of the causes of heart failure, and many of the therapies proven to be of clinical benefit targets at reducing this stress *in vivo*. Drugs that are established to improve the excessive oxidative stress may act indirectly for treatment in heart failure. Treatment with *β*-adrenoceptor blockers like carvedilol and nebivolol reduces generation of ROS by the activation of Jun kinases and stimulation of apoptosis in addition to their antioxidant properties [166, 167]. Statins treatment has irrespective of their cholesterol-lowering activity, leads to reduced G protein signalling response [168]. ACE inhibitors and angiotensin receptor blockers have been shown to reduce BP and markers of oxidative stress in target tissues by reducing the G protein linked signaling to NAD(P)H oxidase. Another drug rosuvastatin also reduces the rate of heart failure significantly [169]. Hydralazine has been demonstrated to reduce mortality in heart failure [170] and has additional inhibitory effects on the generation of superoxide and peroxynitrite [171]. Two other trial drugs, like allopurinol and its active metabolite oxypurinol [172], have been tested extensively in heart failure cases. Other drugs with antioxidant properties, such as probucol [173], edaravone [174] have been used in the treatment of oxidative stress-related diseases. Irbesartan decreases inflammation which contributes to plaque stabilization by inhibition of MMPase-induced plaque rupture [175]. Mevalonate is a precursor for the synthesis of ubiquinone, a natural antioxidant essential for mitochondrial electron chain transport activity, and essential heart function. The use of combination supplement of zinc, ascorbic acid, a-tocopherol, and *β*-carotene has been tested in reduction of systolic and diastolic blood pressure [176]. However, large clinical trials of the antioxidant vitamins (A and E) or their precursors have been disappointing, with no evidence of benefit on cardiac mortality or morbidity.

The discussions *vide supra* advocates the role of thyroid dysfunctions in the pathophysiology of heart diseases mediated by ROS. In a preliminary study reported that the level of LPX increased in the cardiac mitochondria of PTU-treated hypothyroid rats which returned to normal upon T_3_ treatment. On the other hand, mitochondrial SOD activity reduced under hypothyroid condition which was further reduced upon T_3_ supplementation. In contrast, altered thyroid status failed to elicit any change in mitochondrial SOD activity. In case of postmitochondrial fraction, hypothyroid state did not alter the SOD activity, but T_3_ supplementation augmented the enzyme activity. The increased catalase activity observed in postmitochondrial fraction of rat heart came to the level of control upon T_3_ supplementation. The results thus surmise that any alteration in thyroid status may cause subtle changes in cardiac tissue leading to oxidative tissue injury and thyroid hormone replacement therapy is inadequate in bringing back the tissue parameters to normal levels [177]. Albeit antioxidant therapy is not the direct answer to thyroid induced cardiac dysfunction, it promises to minimize tissue damage and accompanying symptoms in this hormonal disorder.

**Figure 3:**
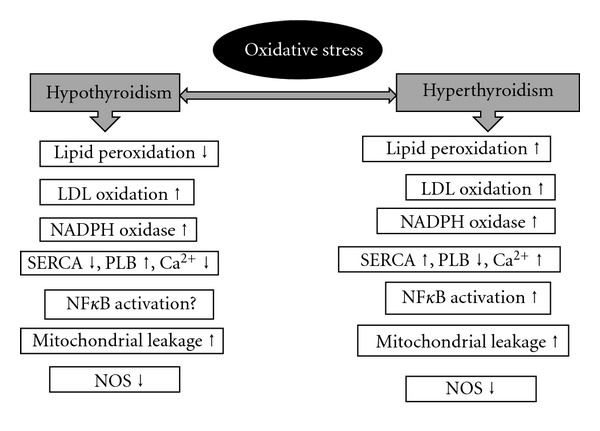
Cardiac oxidative stress under altered thyroid states.

 Altered-thyroid-state-linked changes in the tissues like heart modify their susceptibility to oxidants and the extent of the oxidative damage they suffer following oxidative challenge. Identification of specific TH-responsive molecular pathways via ROS will provide further information related to molecular mechanisms by which changes in the thyroid status alter heart function. However, unrevealing the molecular pathways by which ROS influences cardiac functions is intimidating because of the involvement of multiple signaling pathways and cell targets (Figure 3). Nevertheless understanding few of these pathways may at least provide a plausible road-map of further investigation using animal models to establish a casual link between altered thyroid states induced oxidative stress and heart failure. Futuristic approaches such as antioxidant treatment and gene therapy using viral vectors to modulate the expression of antioxidant genes appear to be a promising strategy to counter oxidative stress-induced myocardial damage.
